# CMR Left Ventricular Filling Pressure Exhibits Strong Haemodynamic Relevance and Outperforms Echocardiography in Multimodal Heart Failure Assessment

**DOI:** 10.3390/jcdd12070250

**Published:** 2025-06-27

**Authors:** Aradhai Bana, Rui Li, Zia Mehmood, Craig Rogers, Ciaran Grafton-Clarke, Tiya Bali, David Hall, Mustapha Jamil, Liandra Ramachenderam, Uwais Dudhiya, Hilmar Spohr, Victoria Underwood, Rebekah Girling, Bahman Kasmai, Sunil Nair, David P. Ripley, Gareth Matthews, Pankaj Garg

**Affiliations:** 1 Norwich Medical School, University of East Anglia, Norwich NR4 7TJ, UK; a.bana@uea.ac.uk (A.B.); r.li2@uea.ac.uk (R.L.); zia.mehmood@uea.ac.uk (Z.M.); ciaran.clarke@nnuh.nhs.uk (C.G.-C.); t.bali@uea.ac.uk (T.B.); gareth.matthews@uea.ac.uk (G.M.); 2 Norfolk and Norwich University Teaching Hospitals, Norwich NR4 7UY, UK; craig.rogers@nnuh.nhs.uk (C.R.); david.hall@nnuh.nhs.uk (D.H.); mustapha.jamil@nnuh.nhs.uk (M.J.); liandra.ramachenderam@nnuh.nhs.uk (L.R.); uwais.dudhiya@nnuh.nhs.uk (U.D.); hilmar.spohr@nnuh.nhs.uk (H.S.); victoria.underwood@nnuh.nhs.uk (V.U.); rebekah.girling@nnuh.nhs.uk (R.G.); b.kasmai@uea.ac.uk (B.K.); sunil.nair@nnuh.nhs.uk (S.N.); 3 Department of Cardiology, Northumbria Healthcare NHS Foundation Trust, Northumbria Specialist Emergency Care Hospital, Cramlington NE29 8NH, UK; david.ripley@northumbria-healthcare.nhs.uk

**Keywords:** N-terminal pro-brain natriuretic peptide, prospective studies, pulmonary capillary wedge pressure, area under curve, sex characteristics, heart failure, echocardiography, biomarkers, heart atria, magnetic resonance imaging

## Abstract

Background: Left ventricular filling pressure (LVFP) is pivotal in heart failure management, yet non-invasive assessment remains challenging. While echocardiography is the first line, cardiovascular magnetic resonance (CMR) offers enhanced accuracy. This study evaluates the interplay between CMR-derived LVFP and echocardiography, focusing on sex differences and correlations with N-terminal pro-brain natriuretic peptide (NT-proBNP). Methods: In this prospective study, 222 patients with CMR-derived LVFP > 14 mmHg underwent transthoracic echocardiography (TTE) and CMR. Sex-specific CMR equations (incorporating left atrial volume and ventricular mass) were used to estimate pulmonary capillary wedge pressure (PCWP). Correlations between imaging parameters and NT-proBNP were assessed. Results: CMR-derived LVFP showed no sex-based differences (*p* = 0.3143), unlike echocardiographic indices: women had higher E/e′ (*p* < 0.0001) and lower lateral mitral annular velocities (*p* = 0.0159). CMR-derived LVFP correlated strongly with NT-proBNP (r = 0.47, *p* < 0.0001), outperforming E/e′ (r = 0.41). Stratification by CMR PCWP tertiles revealed higher NT-proBNP (*p* = 0.0003), left atrial volumes (*p* < 0.0001), and septal thickness (*p* < 0.0001) in the highest tertiles. CMR-derived LVFP demonstrated superior diagnostic accuracy (AUC = 0.754 vs. 0.740 for E/e′) in identifying elevated NT-proBNP (>400 pg/mL). Sex-independent CMR measures contrasted with echocardiography, where parameters like left atrial volume varied by sex (*p* = 0.012). Conclusions: CMR-derived LVFP is a robust, sex-independent biomarker strongly linked to NT-proBNP, offering superior diagnostic performance over echocardiography. Its integration with echocardiographic indices enhances the non-invasive assessment of cardiac filling pressures, advocating a synergistic imaging approach to refine heart failure management.

## 1. Introduction

Left ventricular filling pressure (LVFP) is a critical determinant in the diagnosis, management, and prognostication of heart failure. The American College of Cardiology, American Heart Association, and Heart Failure Society of America guidelines emphasise the importance of assessing LVFP in heart failure patients [1]. Similarly, the European Society of Cardiology guidelines highlight the importance of the assessment of LVFP in heart failure [2,3].

Invasive haemodynamic assessment, such as pulmonary artery catheterisation, is considered the gold standard for measuring LVFP. Non-invasively, echocardiography has been the first-line test for LVFP assessment. However, its modest sensitivity and the lack of robust validation studies limit its reliability when compared with invasive haemodynamic measurements [4]. In recent years, cardiovascular magnetic resonance (CMR) has emerged as a promising alternative, offering enhanced accuracy and reproducibility in quantifying LVFP [5]. Despite these advances, the interplay between CMR-derived LVFP and traditional echocardiographic indices remains poorly understood, creating uncertainty in how best to integrate these complementary modalities into clinical practice. A better understanding of this interplay could inform integrated imaging strategies, ultimately improving clinical decision-making and outcomes in heart failure management.

We hypothesise that LVFP derived from CMR will exhibit sex-independent performance and demonstrate a superior correlation with myocardial stress biomarkers like N-terminal pro-B-type natriuretic peptide (NT-proBNP) compared to traditional assessments. Furthermore, we propose that LVFP measurements obtained via individual echocardiographic parameters will significantly correlate with CMR-derived LVFP, thereby cross-validating the complementary clinical roles of these imaging modalities in cardiac haemodynamic evaluation.

Accordingly, this study aimed to evaluate the association between CMR-derived LVFP and echocardiographic measures to investigate sex-based differences in these imaging indices, and to determine the complementary role of CMR and echocardiography in refining the non-invasive assessment of LVFP in patients with chronically elevated filling pressures.

## 2. Methods

### 2.1. Study Cohort

This study utilised data from the prospective PREFER-CMR registry (ClinicalTrials.gov: NCT05114785) to identify eligible participants. It was designed as an all-comers study, including individuals who underwent transthoracic echocardiography (TTE) followed by CMR imaging between February 2022 and July 2024.

### 2.2. Inclusion Criteria

Participants were required to be at least 18 years of age and to have undergone TTE as part of their diagnostic assessment, followed by a clinically indicated CMR. For the purpose of this focused investigation, only individuals with CMR-derived LVFP exceeding 14 mmHg were included.

### 2.3. Exclusion Criteria

The exclusion criteria encompassed individuals with contraindications to CMR, including those with non-compatible implanted defibrillators, severe claustrophobia, or advanced renal dysfunction (eGFR < 30 mL/min/1.73 m^2^).

### 2.4. Ethical Approval and Consent

This study adhered to the ethical principles outlined in the 1964 Declaration of Helsinki and its subsequent amendments. Ethical approval for data collection and management was obtained from the National Research Ethics Service in the United Kingdom (reference: 21/NE/0149, approved on 21 September 2021). A pragmatic opt-out approach to informed consent was employed for all participants. 

### 2.5. Transthoracic Echocardiography

Standard TTE was performed in accordance with the guidelines set by the British Society of Echocardiography (BSE). The evaluation focused on key parameters essential for assessing LVFP, including the ratio of early diastolic mitral inflow velocity to early diastolic mitral annular velocity (E/e’), the early diastolic mitral annular velocity (e’), tricuspid regurgitation (TR) peak velocity, septal thickness, and left atrial (LA) volume. These echocardiographic indices were systematically measured to provide a comprehensive assessment of diastolic function and ventricular compliance. All echocardiographic measurements were conducted by trained sonographers and reviewed by experienced cardiologists to ensure accuracy and adherence to standardised protocols.

### 2.6. CMR Protocol and Analysis

All CMR studies were performed using a 1.5 Tesla Magnetom Sola system (Siemens Healthineers, Erlangen, Germany) equipped with BioMatrix Body 18 coil technology to enhance imaging precision and patient adaptability. The standardised CMR protocol encompassed baseline localisation surveys followed by cine imaging sequences to assess cardiac structure and function. These cine sequences included vertical long-axis, horizontal long-axis, and short-axis contiguous left ventricular volume stack acquisition. Cine images were obtained during end-expiratory breath-hold using a balanced steady-state free precession (bSSFP) single-slice breath-hold sequence. A total of 30 phase cine images were acquired with a contiguous slice thickness of 8 mm for the short-axis stack. Imaging parameters included an echo time (TE) of 1.13 ms, a repetition time (TR) of 2.71 ms, a flip angle of 80°, a field of view (FOV) of 360 × 289 mm^2^, and a generalised autocalibrating partial parallel acquisition (GRAPPA) acceleration factor of 2.

All imaging procedures were conducted by trained radiographers and analysed by experienced cardiologists and technicians with more than 3 years of CMR experience in accordance with established CMR guidelines to maintain consistency and diagnostic accuracy. Furthermore, CMR data post-processing and interpretation were conducted independently and blinded to echocardiographic findings and other clinical parameters to eliminate potential bias and maintain the integrity of the study results.

CMR image analysis was performed using CVI 42 software (Version 5.17.1, Circle Cardiovascular Imaging, Calgary, AB, Canada). Left ventricular end-diastolic volume (mL), left ventricular end-systolic volume (mL), left ventricular stroke volume (mL), left ventricular ejection fraction (%), left ventricular mass in diastole (g), and left atrial volume at end-systole (mL) were quantified using semi-automated contouring methods on short-axis cine images. Manual adjustments were performed as necessary to ensure precision. Left ventricular volumes and ejection fraction were derived using Simpson’s method of discs. Left ventricular mass was calculated by subtracting the endocardial volume from the epicardial volume and multiplying by myocardial density.

#### Estimation of Left Ventricular Filling Pressure Using Sex-Specific CMR-Derived Equations

To estimate LVFP, we utilised sex-specific equations derived from CMR metrics, as described previously. This equation incorporates left atrial volume (LAV) and left ventricular mass (LVM) to calculate the pulmonary capillary wedge pressure (PCWP), serving as a surrogate for LVFP [6]. LAV was measured using the biplane area-length method from the 2- and 4-chamber cine images at end-systole. Left ventricular mass was determined through short-axis segmentation in end-diastole using established techniques. Papillary muscles were included in blood volume. These parameters were integrated into sex-specific equations to derive estimates of LVFP, providing a robust method for evaluating diastolic function. The sex-specific equation is as follows:

CMR PCWP = 5.7591 + (0.07505 × LAV) + (0.05289 × LVM) − (1.9927 × sex) [female = 0; male = 1]

where

- PCWP is the pulmonary capillary wedge pressure in mmHg.
- LAV is the left atrial volume in mL.
- LVM is the left ventricular mass in g.

All CMR image analyses were conducted using dedicated research software (MASS version 2021-Exp, Leiden University Medical Center, Leiden, The Netherlands). CMR contour tracings, including volume/function assessments and late gadolinium enhancement (LGE) segmentation, were performed by R.J.G at core-lab (Leiden).

### 2.7. Statistical Analysis

A cross-sectional observational design was employed. Data are presented as mean ± standard deviation. Between-group comparisons of continuous variables were performed with the Mann–Whitney U test (for two groups) or the Kruskal–Wallis test (for three or more groups). Categorical variables were expressed as counts and percentages, and differences were examined using the chi-square test. When global tests were significant, post hoc pairwise comparisons (e.g., Dunn’s test) were applied with appropriate corrections for multiple testing. Correlations between continuous variables (e.g., echocardiographic indices and CMR-derived LVFP or NT-proBNP) were evaluated using Spearman’s rank correlation coefficient. Receiver operating characteristic (ROC) curve analyses were performed to assess diagnostic performance, and the area under the curve (AUC) was calculated to compare the discriminative power of CMR LVFP and echocardiographic parameters. To assess for interaction between sex and E/e′ ratio in predicting CMR-derived LVFP, we performed a least squares multiple linear regression analysis with LVFP as the dependent variable and sex, E/e′ ratio, and their interaction term (sex × E/e′) as independent variables. The interaction term was manually generated by multiplying sex and E/e′ values. The statistical significance was evaluated based on the *p*-value for the interaction coefficient, with a threshold of *p* < 0.05 indicating significant effect modification. Statistical significance was set at *p* < 0.05 for all tests. Statistical analyses were conducted with IBM SPSS Statistics (version 23) and MedCalc (Version 23.1.6).

### 2.8. Sample Size Determination

Based on previous studies [6], we determined that detecting a 2 mmHg difference in CMR-derived LV filling pressure—considered clinically significant—with an assumed standard deviation of 3 mmHg, a two-sided alpha of 0.05, and 80% power would require approximately 70 subjects per group. This translates to a minimum total sample size of 210 patients for the three tertile groups. Additionally, for sex-based comparisons using an independent samples *t*-test with a medium effect size (Cohen’s d ≈ 0.5), roughly 64 subjects per group would be required. With a total enrolment of 222 patients (60% male), our study was sufficiently powered to detect meaningful differences in both CMR and echocardiographic parameters.

## 3. Results

### 3.1. Study Population

A total of 222 patients with high CMR-derived LVFP were recruited to the study with a mean age of 57.7 years, and 60% were men (Table 1). There was a median 4-month difference between TTE and CMR assessment. Male participants were taller and heavier than females (both *p* < 0.0001) and exhibited higher creatinine and haemoglobin levels (both *p* < 0.0001). The estimated glomerular filtration rate trended higher in men (*p* = 0.0594), although NT-proBNP did not differ significantly by sex (*p* = 0.1654). On echocardiography, men showed a larger absolute left atrial volume (*p* = 0.012), whereas late diastolic mitral flow velocity was higher in females (*p* = 0.0007). Notably, women had a significantly higher E/e′ ratio (*p* < 0.0001), suggesting a tendency toward elevated left ventricular filling pressures despite no statistical difference in the CMR-derived filling pressure itself (*p* = 0.3143). In a multiple linear regression model including sex, E/e′ ratio, and their interaction term (sex × E/e′), the interaction was not statistically significant (*p* = 0.248), indicating that sex did not materially modify the relationship between E/e′ and LVFP.

In contrast, the CMR findings revealed significantly larger left ventricular end-diastolic, end-systolic, and stroke volumes in men (all *p* < 0.0001). Men also had higher left ventricular mass (*p* < 0.0001), while women demonstrated a mildly higher ejection fraction (*p* = 0.0091). Interventricular septum thickness was significantly greater in men (*p* = 0.0053), indicating more pronounced structural remodelling. Overall, these data highlight important sex-based variations in both echocardiographic and CMR-derived measures, with the most pronounced differences in anthropometrics, ventricular volumes, and myocardial mass.

### 3.2. CMR-Derived PCWP Stratification

**Figure 1:**
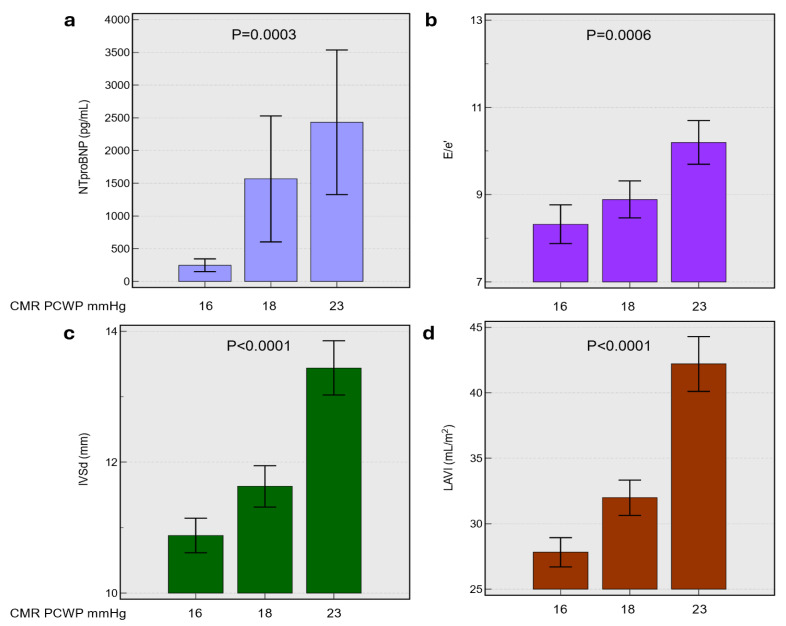
Relationship between CMR-derived LVFP or pulmonary capillary wedge pressure and key cardiac biomarkers including panel (**a**), N-terminal pro-brain natriuretic peptide (NT-proBNP), (**b**) ratio of early mitral inflow velocity to early diastolic annular velocity (E/e’), (**c**) interventricular septal thickness in diastole (IVSd), and (**d**) left atrial volume index (LAVI) across mean CMR PCWP values of 16, 18, and 23 mmHg. Bar graphs display mean ± standard deviation. Statistical significance was determined by ANOVA, with *p*-values indicated. Abbreviations: CMR, cardiac magnetic resonance; PCWP, pulmonary capillary wedge pressure; NT-proBNP, N-terminal pro-brain natriuretic peptide; E/e’, ratio of early mitral inflow velocity to early diastolic annular velocity; IVSd, interventricular septal thickness in diastole; LAVI, left atrial volume index.

The participants were stratified into three tertiles based on CMR-derived PCWP: T1 (16.1 ± 0.6 mmHg), T2 (18.1 ± 0.6 mmHg), and T3 (22.7 ± 3.1 mmHg). The baseline characteristics were broadly comparable across tertiles in age, sex distribution, renal function, and haemoglobin (all *p* > 0.05) (Table 2). However, the highest PCWP tertiles (T3) exhibited a significantly higher mean body weight compared to T1 (*p* < 0.0001). In terms of past medical history, T3 showed a higher prevalence of hypertension (*p* = 0.0022) and atrial fibrillation (*p* = 0.0365), whereas myocardial infarction was more frequent in T1 (*p* = 0.0028). Notably, NT-proBNP levels were markedly elevated in T2 and T3 relative to Q1 (*p* = 0.0003) (Figure 1).

The echocardiographic and CMR assessments demonstrated a stepwise increase in left atrial volumes (indexed and absolute) across the CMR-derived PCWP tertiles, culminating in significantly larger volumes in T3 (*p* < 0.0001) (Figure 1). The E/e′ ratio was also significantly higher in T3 (10.2 ± 4.4) compared to T1 (8.3 ± 3.8) (*p* = 0.0006), while lateral and septal mitral annular velocities were lower in T3 (*p* < 0.01). Likewise, left ventricular end-diastolic volume and stroke volume increased from T1 to T3 (*p* ≤ 0.002), and LV mass in diastole was substantially greater in T3 compared with T1 (*p* < 0.0001). Despite these volumetric changes, no significant differences were observed in LV ejection fraction across the three tertiles.

**Figure 2:**
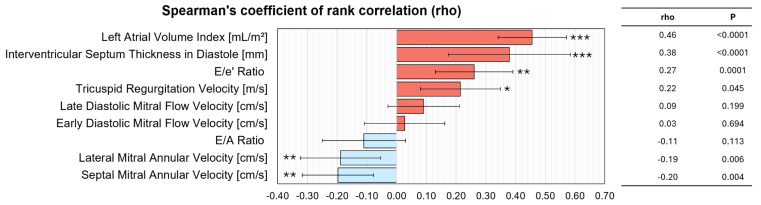
Correlation between CMR-derived LVFP and echocardiographic indices. The bar graph illustrates Spearman’s rank correlation coefficients (rho) with 95% confidence intervals for key echocardiographic parameters, ranked by correlation strength. Positive correlations (red) indicate increasing values with higher LVFP, while negative correlations (blue) indicate decreasing values. Statistical significance is denoted as *p* < 0.05 (*), *p* < 0.01 (**), and *p* < 0.001 (***). Abbreviations: CMR, cardiac magnetic resonance; LVFP, left ventricular filling pressure; LAVI, left atrial volume index; IVSd, interventricular septal thickness in diastole; E/e’, ratio of early mitral inflow velocity to early diastolic annular velocity; TRV, tricuspid regurgitation velocity; A, late diastolic mitral flow velocity; E, early diastolic mitral flow velocity; E/A, ratio of early to late diastolic mitral flow velocity; Lateral e’, lateral mitral annular velocity; Septal e’, septal mitral annular velocity.

Spearman’s rank correlation analysis of echocardiographic parameters against CMR-derived LV filling pressure revealed that left atrial volume index and interventricular septum thickness in diastole exhibited the strongest positive associations (both *p* < 0.0001), followed by significant correlations for E/e′ ratio (*p* = 0.0003) and tricuspid regurgitation velocity (*p* = 0.002) (Figure 2). Early and late diastolic mitral flow velocities, as well as the ratio of early to late diastolic mitral flow velocity (E/A), did not correlate significantly. In contrast, lateral and septal mitral annular velocities were inversely related to CMR LV filling pressure (*p* < 0.01 for both), suggesting that higher annular velocities align with lower filling pressures, while increased left atrial volume and septal thickness reflect elevated filling pressures.

### 3.3. NTproBNP-Based Stratification

Echocardiographic measurements demonstrated a significant rise in left atrial size (both indexed and absolute) with increasing NTproBNP, along with marked elevations in E/e′ ratio (*p* = 0.0015) and tricuspid regurgitation velocity (*p* = 0.0092) (Table 3). Interventricular septum thickness was also significantly greater in the highest NTproBNP category (*p* = 0.0053), while mitral inflow (E/A ratio) did not vary appreciably. These findings suggest that as NTproBNP levels rise, there is a parallel increase in both left- and right-sided filling pressures, reflecting heightened cardiac workload and remodelling. On CMR, the left ventricular ejection fraction declined significantly in those with the highest NTproBNP (*p* = 0.0145), and LV mass in the diastole showed a notable upward trend (*p* = 0.0432). Although LV volumes did not differ substantially across groups, the CMR-derived LV filling pressure displayed the strongest between-group contrast (*p* = 0.0002).

**Figure 3:**
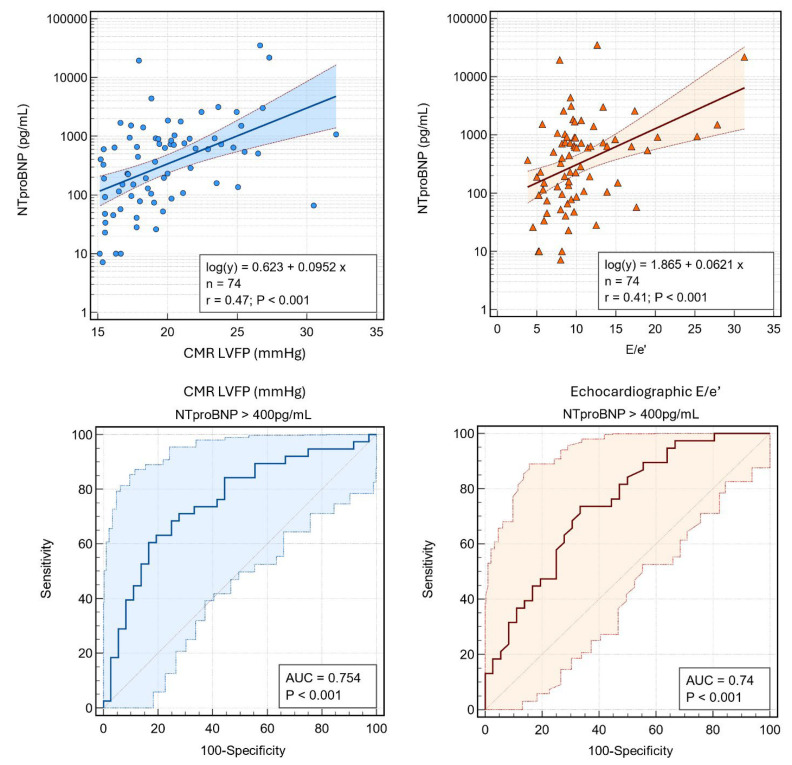
Associations between NT-proBNP with CMR-derived LVFP and echocardiographic E/e’. (**Top**) Scatter plots with logarithmic regression lines and 95% confidence intervals show correlations of NT-proBNP with (**left**) CMR LVFP (r = 0.47, *p* < 0.001) and (**right**) E/e’ (r = 0.41, *p* < 0.001). (**Bottom**) Receiver operating characteristic (ROC) curves illustrate the diagnostic accuracy of (**left**) CMR LVFP and (**right**) E/e’ for predicting NT-proBNP >400 pg/mL. Abbreviations: NT-proBNP, N-terminal pro-brain natriuretic peptide; CMR, cardiac magnetic resonance; LVFP, left ventricular filling pressure; E/e’, ratio of early mitral inflow velocity to early diastolic annular velocity; AUC, area under the curve.

CMR-derived LV filling pressure showed a strong correlation with NTproBNP (r = 0.47, *p* < 0.0001), whereas the E/e′ ratio exhibited a more modest yet significant relationship (r = 0.41, *p* < 0.0001) (Figure 3). In discriminating patients with elevated NTproBNP (>400 pg/mL), CMR LV filling pressure demonstrated higher diagnostic accuracy (AUC = 0.75) compared to the E/e′ ratio (AUC = 0.74). These findings underscore the robust physiological link between CMR-derived LV filling pressure and natriuretic peptide levels, highlighting its potential superiority for assessing elevated cardiac filling pressures. Collectively, these results underscore the complementary nature of echocardiography and CMR, with CMR-derived filling pressure emerging as the most sensitive parameter in discriminating patients based on NTproBNP levels.

In stepwise least-squares multiple regression, left ventricular filling pressure was the sole independent predictor of NT-proBNP concentration. The final model accounted for 26.5% of the variance (R^2^ = 0.26; adjusted R^2^ = 0.23), with a multiple correlation coefficient of 0.52 and a residual standard deviation of 678 pg mL^−1^. Each 1 mmHg increase in left ventricular filling pressure corresponded to an estimated 108 pg mL^−1^ rise in NT-proBNP (95% CI 24–193 pg mL^−1^; t = 2.69; *p* = 0.014), and the partial correlation coefficient for this association was 0.515. The model intercept was −1515 pg mL^−1^ (95% CI −3199 to 169 pg mL^−1^; *p* = 0.08). The ratio of early transmitral flow velocity to early diastolic mitral annular velocity, left atrial volume index, tricuspid regurgitation velocity, and the ratio of early to late transmitral flow velocity failed to satisfy the entry criterion (*p* < 0.05) and were excluded from the multivariable model.

## 4. Discussion

Our study demonstrates that CMR-derived LVFP is remarkably consistent across sexes, in contrast to several echocardiographic LVFP indices that show significant sex-based differences. Specifically, while echo parameters such as the E/e′ ratio and left atrial volumes vary between men and women, CMR-derived LVFP remains unaffected by sex, underscoring its robustness as a physiological marker. Moreover, CMR-derived LVFP exhibited the strongest association with NT-proBNP levels, reinforcing its superior link to cardiac stress and underlying pathophysiology. Importantly, these results do not diminish the value of echocardiography; rather, the significant interplay between echo-derived indices and CMR PCWP highlights the complementary strengths of these modalities. Together, our findings advocate for an integrated imaging approach that leverages the physiological fidelity of CMR-derived LVFP alongside the accessibility and broad applicability of echocardiographic assessments.

Our findings build upon previous validations of the CMR-based equation for estimating LVFP [5,6] both of which demonstrated strong correlations with invasive hemodynamic measurements. Notably, those initial investigations reported that CMR-derived LVFP outperforms conventional echocardiographic surrogates in mirroring true filling pressures. Consistent with these observations, our study underscores the robust physiological linkage between CMR LVFP and NT-proBNP, highlighting its clinical potential as a non-invasive marker of diastolic burden. Furthermore, we observed that CMR LVFP remains unaffected by sex—an important distinction given that multiple echocardiographic parameters in our cohort varied between men and women. This suggests that CMR-based assessment may offer greater consistency across diverse patient populations, reinforcing its value as a standardised measure of cardiac filling pressures.

Despite these advantages, echocardiography remains indispensable as a first-line modality, thanks to its accessibility and established role in routine cardiac assessment. Indeed, several echo-derived indices (e.g., E/e′, left atrial volume) did correlate significantly with CMR LVFP in our study, reflecting their complementary strengths in detecting diastolic dysfunction. In light of the earlier validations showing superior concordance between CMR and invasive measures, the synergy between CMR and echocardiography becomes even more compelling—particularly in borderline or complex cases where a single modality might not provide a complete picture. Taken together, our results expand upon the foundational work of previous investigations, demonstrating that CMR-derived LVFP remains robust across varying patient characteristics and augments the diagnostic yield of standard echocardiography. Future prospective multicentre trials are warranted to fully integrate these findings into routine clinical algorithms and to evaluate their impact on patient outcomes.

**Figure 4:**
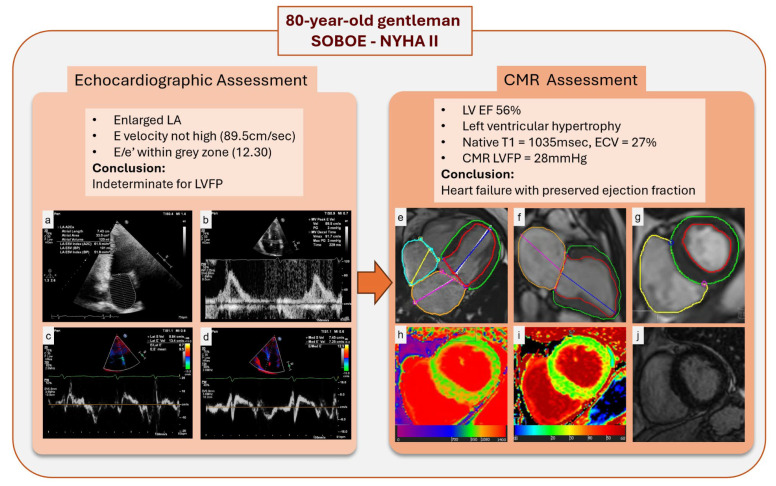
Multimodal assessment of LVFP and myocardial characteristics in an 80-year-old male with shortness of breath on exertion and NYHA class II symptoms. Panel (**a**–**d**): Echocardiographic assessment demonstrates an enlarged LA, an E velocity of 89.5 cm/sec, and an E/e’ ratio of 12.3, placing LVFP in an indeterminate range. Panel (**e**–**g**): CMR showed left ventricular hypertrophy and preserved LVEF, similar to echocardiography. (**h**,**i**): Native T1 mapping and extracellular volume (ECV) fraction mapping reveal regions of increased values, suggesting subtle myocardial fibrosis. (**j**): Late gadolinium enhancement (LGE) imaging shows no noticeable scar. Abbreviations: SOBOE, shortness of breath on exertion; NYHA, New York Heart Association; LA, left atrium; LVFP, left ventricular filling pressure; LVEF, left ventricular ejection fraction; CMR, cardiac magnetic resonance; ECV, extracellular volume; LGE, late gadolinium enhancement.

In comparing our findings with those of Assadi et al. [7], both studies used a validated CMR-derived LV filling pressure equation [5,6] and demonstrated a strong independent association with NT-proBNP. However, while the previous study evaluated a generic heart failure population regardless of LVFP levels, our study focused specifically on patients with high LVFP by CMR. Moreover, our comprehensive echocardiographic data revealed significant sex-based differences in measures such as the E/e′ ratio and left atrial volume, whereas CMR-derived LVFP remained robust and sex-independent. These results underscore the superior physiological link of CMR LVFP with NT-proBNP and its incremental diagnostic and prognostic value (Figure 4), supporting its integration into routine clinical practice alongside echocardiography.

Moreover, our previous work has demonstrated that adenosine stress acutely increases left atrial volume and CMR-derived LV filling pressure using a validated equation [8]. In contrast, our current work in patients with chronically elevated LVFP revealed significant sex differences in echocardiographic parameters, while CMR LVFP remained robust and sex-independent, strongly correlating with NT-proBNP and underscoring its clinical utility alongside echocardiography. Another recent UK Biobank study [9] highlighted that raised CMR-modelled LVFP independently predicts incident heart failure and major adverse cardiovascular events in the general population. Even though our work did not look at hard outcomes, in conjunction with previous work from the UK Biobank, it is evident that CMR-derived LVFP is primed for clinical translation.

### Limitations

This study is limited by its cross-sectional observational design, which precludes assessment of longitudinal outcomes. Although causal relationships cannot be definitively established, the robust associations observed between CMR-derived LV filling pressure, NT-proBNP levels, and echocardiographic indices are in line with previous studies, supporting the relevance of our findings. Additionally, the single-centre recruitment of 222 patients may constrain generalisability; however, the demographic and clinical profiles of our cohort mirror real-world practice, enhancing external validity. Another limitation is the inherent variability in echocardiographic measurements, which are operator-dependent and influenced by patient-specific factors. In contrast, CMR offers high reproducibility and the precise quantification of LV filling pressures despite being more costly and less widely available. Despite these challenges, the significant interplay between CMR and echocardiographic indices reinforces the complementary value of a multimodal imaging approach. Moreover, while NT-proBNP levels can be affected by comorbid conditions, their strong correlation with CMR-derived LV filling pressure underscores the physiological relevance of our findings. These limitations, when balanced against the study’s strengths, support the potential for integrating CMR into routine clinical practice to enhance diagnostic precision.

## 5. Conclusions

CMR-derived PCWP is a robust, sex-independent biomarker of LVFP that strongly correlates with NT-proBNP and warrants routine clinical use. Its complementary role with echocardiography supports an integrated imaging approach for enhanced diagnostics and patient care.

## Figures and Tables

**Table 1:** Patient demographics stratified by sex.

	Male	Female	*p* ^#^
	n = 134	n = 88	
Age [years]	56.3 ± 16.3	59.9 ± 15.2	0.1095
Height [cm]	179.7 ± 26.2	163.1 ± 6.1	<0.0001
Weight [kg]	90.5 ± 16.8	79.4 ± 19.4	<0.0001
Estimated Glomerular Filtration Rate [mL/min/1.73 m^2^]	80.6 ± 13.0	76.2 ± 17.3	0.0594
Creatinine [mg/dL]	88.6 ± 25.6	80.9 ± 68.1	<0.0001
Haemoglobin [g/dL]	145.6 ± 14.2	135.6 ± 13.6	<0.0001
N-Terminal Pro-B-Type Natriuretic Peptide [pg/mL]	1080.2 ± 2972.0	2526.1 ± 7281.0	0.1654
Echocardiographic Assessment			
Left Atrial Volume Index [mL/m^2^]	34.7 ± 15.1	33.6 ± 10.8	0.9772
Left Atrial Volume [mL]	70.5 ± 28.3	60.0 ± 16.0	0.012
Early Diastolic Mitral Flow Velocity [cm/s]	1.5 ± 6.7	0.7 ± 0.2	0.0898
Late Diastolic Mitral Flow Velocity [cm/s]	2.0 ± 11.8	2.1 ± 11.8	0.0007
E/A Ratio	1.1 ± 0.4	1.0 ± 0.4	0.1499
E/e’ Ratio	8.1 ± 3.0	10.5 ± 4.9	<0.0001
Lateral Mitral Annular Velocity [cm/s]	10.4 ± 3.8	9.0 ± 3.6	0.0159
Septal Mitral Annular Velocity [cm/s]	8.0 ± 2.8	7.3 ± 2.6	0.0732
Tricuspid Regurgitation Velocity [m/s]	2.3 ± 0.5	2.4 ± 0.5	0.3444
Interventricular Septum Thickness in Diastole [mm]	12.4 ± 3.2	11.3 ± 2.5	0.0053
CMR Assessment			
Left Ventricular End-Diastolic Volume [mL]	166.6 ± 32.1	141.1 ± 32.8	<0.0001
Left Ventricular End-Systolic Volume [mL]	65.9 ± 18.5	52.1 ± 19.2	<0.0001
Left Ventricular Stroke Volume [mL]	100.8 ± 20.9	88.6 ± 19.8	<0.0001
Left Ventricular Ejection Fraction [%]	0.6 ± 0.08	0.6 ± 0.08	0.0091
Left Ventricular Mass in Diastole [g]	162.2 ± 44.1	124.1 ± 36.8	<0.0001
Left Ventricular Filling Pressure [mmHg]	19.3 ± 3.7	18.4 ± 2.6	0.3143

Data are mean ± standard deviation. ^#^ Mann–Whitney test.

**Table 2:** CMR characteristics stratified by CMR PCWP tertiles.

CMR-Derived LVFP	Tertile 1	Tertile 2	Tertile 3	*p*-Value
	n = 74	n = 74	n = 74	
CMR-derived LVFP [mmHg]	16.1 ± 0.6 (a)(b)	18.1 ± 0.6 (a)	22.7 ± 3.1 (b)	<0.0001 ^#^
Age [years]	56.7 ± 15.1	55.5 ± 18.0	61.1 ± 14.1	0.1350 ^#^
Height [cm]	173.1 ± 11.2	174.8 ± 35.7	171.4 ± 9.3	0.5293 ^#^
Weight [kg]	81.8 ± 15.3 (a)	82.7 ± 17.5	93.7 ± 20.5 (a)	0.0001 ^#^
Male N(%)	47 (64%)	38 (51%)	49 (66%)	0.1438 ^§^
Estimated Glomerular Filtration Rate [mL/min/1.73 m^2^]	78.8 ± 13.5	80.9 ± 14.2	76.9 ± 17.0	0.2021 ^#^
Creatinine [mg/dL]	82.2 ± 18.4	80.8 ± 33.5	93.6 ± 72.1	0.0916 ^#^
Haemoglobin [g/dL]	143.1 ± 13.3	141.3 ± 12.6	140.6 ± 18.0	0.5505 ^#^
N-Terminal Pro-B-Type Natriuretic Peptide [pg/mL]	247.0 ± 411.0 (a)(b)	1567.0 ± 4298.0 (a)	1500.9 ± 3648.0 (b)	0.0003 ^#^
Systolic Blood Pressure [mmHg]	133.7 ± 20.5	130.7 ± 18.2	141.5 ± 23.5	0.0100 ^#^
Diastolic Blood Pressure [mmHg]	79.4 ± 12.1	78.7 ± 12.3	82.3 ± 14.0	0.3166 ^#^
Past Medical History				
Hypertension N (%)	25 (34%) 49 (66%)	23 (31%) 51 (69%)	42 (57%) 32 (43%)	0.0022 ^§^
Diabetes Mellitus N (%)	10 (14%)	11 (15%)	11 (15%)	0.9641 ^§^
Current Smoker N (%)	11 (15%)	6 (8%)	3 (4%)	0.0677 ^§^
Cerebrovascular Accident N (%)	1 (1%)	1 (1%)	5 (7%)	0.0944 ^§^
Atrial Fibrillation N (%)	4 (5%)	8 (11%)	14 (19%)	0.0365 ^§^
Myocardial Infarction N (%)	21 (28%)	6 (8%)	10 (14%)	0.0028 ^§^
Ventricular Tachycardia N (%)	1 (1%)	1 (1%)	2 (3%)	0.7752 ^§^
Echocardiographic Assessment				
Left Atrial Volume Index [mL/m^2^]	27.8 ± 8.1 (a)(b)	32.0 ± 10.3 (a)	42.2 ± 16.4 (b)	<0.0001 ^#^
Left Atrial Volume [mL]	54.5 ± 15.1 (a)	60.2 ± 13.6	83.3 ± 30.6 (a)	<0.0001 ^#^
Early Diastolic Mitral Flow Velocity [cm/s]	1.2 ± 4.0	0.7 ± 0.2	1.7 ± 8.1	0.9820 ^#^
Late Diastolic Mitral Flow Velocity [cm/s]	1.3 ± 4.7	2.2 ± 12.7	2.8 ± 15.9	0.1813 ^#^
E/A Ratio	1.1 ± 0.4	1.1 ± 0.5	1.0 ± 0.4	0.1011 ^#^
E/e’ Ratio	8.3 ± 3.8 (a)	8.7 ± 3.5	10.2 ± 4.4 (a)	0.0006 ^#^
Lateral Mitral Annular Velocity [cm/s]	10.4 ± 3.6 (a)	10.4 ± 4.2	8.7 ± 3.1 (a)	0.0072 ^#^
Septal Mitral Annular Velocity [cm/s]	8.3 ± 2.6 (a)	7.7 ± 2.8	7.0 ± 2.6 (a)	0.0160 ^#^
Tricuspid Regurgitation Velocity [m/s]	2.3 ± 0.5	2.2 ± 0.4	2.5 ± 0.5	0.0157 ^#^
Interventricular Septum Thickness in Diastole [mm]	10.9 ± 2.2 (a)	11.6 ± 2.6	13.4 ± 3.5 (a)	<0.0001 ^#^
CMR Assessment				
Left Ventricular End-Diastolic Volume [mL]	144.7 ± 24.0 (a)(b)	158.7 ± 33.0 (a)	166.1 ± 40.0 (b)	0.0018 ^#^
Left Ventricular End-Systolic Volume [mL]	57.6 ± 15.3	60.8 ± 20.6	62.9 ± 22.9	0.4467 ^#^
Left Ventricular Stroke Volume [mL]	86.7 ± 14.7 (a)(b)	97.8 ± 18.0 (a)	103.3 ± 26.2 (b)	<0.0001 ^#^
Left Ventricular Ejection Fraction [%]	0.6 ± 0.06	0.6 ± 0.07	0.6 ± 0.1	0.3285 ^#^
Left Ventricular Mass in Diastole [g]	123.2 ± 21.0 (a)(b)	135.7 ± 25.9 (a)	182.4 ± 55.9 (b)	<0.0001 ^#^
Left Atrial Volume at End-Systole [mL]	67.4 ± 11.0 (a)(b)	82.1 ± 13.6 (a)	114.3 ± 35.1 (b)	<0.0001 ^#^

Data are mean ± standard deviation, ^#^ Kruskal–Wallis test, ^§^ chi-squared test, letters in brackets depict data which are significantly different.

**Table 3:** TTE and CMR imaging characteristics stratified by NTproBNP levels.

	NTproBNP < 400	NTproBNP ≤ 2000	NTproBNP > 2000	*p* -Value ^#^
Echocardiographic Assessment				
Left Atrial Volume Index [mL/m^2^]	31.7 ± 9.5 (a)	40.4 ± 16.4 (a)	51.2 ± 25.7	0.0448
Left Atrial Volume [mL]	63.2 ± 21.1 (a)	76.8 ± 28.5	90.1 ± 33.5 (a)	0.0476
Early Diastolic Mitral Flow Velocity [cm/s]	0.7 ± 0.2	3.2 ± 12.8	0.8 ± 0.4	0.3203
Late Diastolic Mitral Flow Velocity [cm/s]	0.8 ± 0.2	6.0 ± 25.2	0.9 ± 0.3	0.7239
E/A Ratio	0.9 ± 0.4	10. ± 0.4	0.8 ± 0.4	0.8369
E/e’ Ratio	8.4 ± 2.9 (a)(b)	11.9 ± 5.4 (a)	13.7 ± 7.8 (b)	0.0015
Lateral Mitral Annular Velocity [cm/s]	9.7 ± 2.8	8.3 ± 3.1	8.0 ± 2.9	0.165
Septal Mitral Annular Velocity [cm/s]	7.4 ± 2.6	6.4 ± 3.1	6.6 ± 2.9	0.1815
Tricuspid Regurgitation Velocity [m/s]	2.3 ± 0.3 (a)(b)	2.7 ± 0.7 (a)	3.0 ± 0.4 (b)	0.0092
Interventricular Septum Thickness in Diastole [mm]	11.7 ± 2.9 (a)(b)	13.8 ± 3.8 (a)	15.0 ± 0.8 (b)	0.0053
CMR Assessment				
Left Ventricular End-Diastolic Volume [mL]	156.7 ± 29.1	144.1 ± 36.4	166.4 ± 51.7	0.2021
Left Ventricular End-Systolic Volume [mL]	59.0 ± 16.6	56.0 ± 22.0	75.8 ± 27.6	0.1987
Left Ventricular Stroke Volume [mL]	97.5 ± 19.6	88.2 ± 22.4	90.6 ± 27.5	0.1551
Left Ventricular Ejection Fraction [%]	0.6 ± 0.07 (a)	0.6 ± 0.09	0.6 ± 0.05 (a)	0.0145
Left Ventricular Mass in Diastole [g]	142.5 ± 36.9 (a)	166.1 ± 61.8	192.0 ± 57.8 (a)	0.0432
Left Atrial Volume at End-Systole [mL]	84.8 ± 27.2	95.5 ± 32.3	118.7 ± 56.2	0.0808
CMR-derived Left Ventricular Filling Pressure [mmHg]	18.3 ± 3.1 (a)(b)	20.7 ± 3.7 (a)	23.6 ± 3.6 (b)	0.0002

Data are mean (standard deviation), ^#^ Kruskal–Wallis test, letters in brackets depict data which are significantly different.

## Data Availability

The data underlying this article will be shared by the corresponding author upon reasonable request.

## Abbreviations

CMR	cardiac magnetic resonance
E/A	ratio of early to late diastolic mitral flow velocity
E/e’	ratio of early mitral inflow velocity to early diastolic annular velocity
LAV	left atrial volume
LVM	left ventricular mass
LVFP	left ventricular filling pressure
NT-proBNP	N terminal pro-brain natriuretic peptide
PCWP	pulmonary capillary wedge pressure
TTE	transthoracic echocardiography
